# Supplementary material for: Machine Learning Accelerates Crystallization for Structure Determination
Source: Angew Chem Int Ed Engl. 2026 May 4;65(25):e1218503. doi: 10.1002/anie.1218503 (PMC13266958; doi:10.1002/anie.1218503)

checkCIF/PLATON report

Structure factors have been supplied for datablock(s) lcz062

THIS REPORT IS FOR GUIDANCE ONLY. IF USED AS PART OF A REVIEW PROCEDURE FOR PUBLICATION, IT SHOULD NOT REPLACE THE EXPERTISE OF AN EXPERIENCED CRYSTALLOGRAPHIC REFEREE.

No syntax errors found. CIF dictionary Interpreting this report

Datablock: lcz062

Bond precision:	C-C = 0.0146 A		Wavelength=1.54184
Cell:	a=8.8270(3)	b=12.9600(5)	c=14.3646(6)
	alpha=103.546(3)	beta=104.763(3)	gamma=107.510(3)
Temperature:	100 K		

	Calculated	Reported
Volume	1426.69(11)	1426.69(10)
Space group	P -1	P -1
Hall group	-P 1	-P 1
Moiety formula	C15 H3 Ag3 F18 N6, 0.5(C14 H8 O2)	C15 H3 Ag3 F18 N6, C7 H4 O
Sum formula	C22 H7 Ag3 F18 N6 O	C22 H7 Ag3 F18 N6 O
Mr	1036.95	1036.95
Dx,g cm-3	2.414	2.414
Z	2	2
Mu (mm-1)	17.788	17.788
F000	984.0	984.0
F000’	988.94	
h,k,lmax	11,16,18	11,16,17
Nref	6131	5769
Tmin,Tmax	0.133,0.073	0.544,1.000
Tmin’	0.021	

Correction method= # Reported T Limits: Tmin=0.544 Tmax=1.000
AbsCorr = MULTI-SCAN

Data completeness= 0.941   Theta(max)= 78.267

R(reflections)= 0.0724(4788)   wR2(reflections)= 0.1843(5769)

S = 1.090   Npar= 412

The following ALERTS were generated. Each ALERT has the format
test-name_ALERT_alert-type_alert-level.
Click on the hyperlinks for more details of the test.

Alert level C

```
PLAT042_ALERT_1_C Calc. and Reported MoietyFormula Strings  Differ     Please Check
              Calc: C15 H3 Ag3 F18 N6, 0.5(C14 H8 O2)
              Rep.: C15 H3 Ag3 F18 N6, C7 H4 O
PLAT213_ALERT_2_C Atom F00O            has ADP max/min Ratio .....        3.7 prolat
PLAT213_ALERT_2_C Atom F00P            has ADP max/min Ratio .....        3.3 prolat
PLAT213_ALERT_2_C Atom F012            has ADP max/min Ratio .....        3.4 prolat
PLAT213_ALERT_2_C Atom F01C            has ADP max/min Ratio .....        3.2 prolat
PLAT213_ALERT_2_C Atom C018            has ADP max/min Ratio .....        3.1 oblate
PLAT220_ALERT_2_C NonSolvent   Resd 1  F   Ueq(max)/Ueq(min) Range        4.3 Ratio
PLAT342_ALERT_3_C Low Bond Precision on  C-C Bonds ..............    0.01455 Ang.
PLAT362_ALERT_2_C Short  C(sp3)-C(sp2) Bond  C00Z     - C018     .       1.41 Ang.
PLAT906_ALERT_3_C Large K Value in the Analysis of Variance ......      9.146 Check
PLAT906_ALERT_3_C Large K Value in the Analysis of Variance ......      2.230 Check
PLAT911_ALERT_3_C Missing FCF Refl Between Thmin & STh/L=    0.600         56 Report
                0  1  0,   3  9  2,   4  9  2,  -6  2  3,   4 -8  4,   6 -3  4,
                9 -3  4,   8  0  4,   8 -7  5,   8 -2  5,   8 -1  5,   8 -3  6,
                8 -2  6,   1  1  6, -10  7  6,   8 -5  7,   7 -4  7,   8 -4  7,
                6 -2  7,   6 -1  7,   5  0  7,  -2 10  7,   7 -4  8,  -1 -3  8,
                6 -2  8,   7 -2  8,   5 -1  8,   5  0  8,   4-13  9,   7 -9  9,
                   (   26 More Missing: see the .ckf listing file)
PLAT971_ALERT_2_C Check Calcd Resid. Dens.  1.02Ang From Ag02          2.32 eA-3
PLAT971_ALERT_2_C Check Calcd Resid. Dens.  1.01Ang From Ag01          2.13 eA-3
PLAT971_ALERT_2_C Check Calcd Resid. Dens.  0.88Ang From Ag01          1.81 eA-3
PLAT971_ALERT_2_C Check Calcd Resid. Dens.  0.94Ang From Ag02          1.80 eA-3
PLAT971_ALERT_2_C Check Calcd Resid. Dens.  0.99Ang From Ag03          1.80 eA-3
PLAT971_ALERT_2_C Check Calcd Resid. Dens.  0.33Ang From C00Z          1.68 eA-3
PLAT971_ALERT_2_C Check Calcd Resid. Dens.  0.28Ang From C00W          1.66 eA-3
PLAT972_ALERT_2_C Check Calcd Resid. Dens.  0.97Ang From Ag03         -1.61 eA-3
PLAT972_ALERT_2_C Check Calcd Resid. Dens.  0.76Ang From Ag02         -1.61 eA-3
```

Alert level G

```
PLAT003_ALERT_2_G Number of Uiso or U(i,j)  Restrained non-H-Atoms          1 Report
PLAT083_ALERT_2_G SHELXL Second Parameter in WGHT  Unusually Large      64.35 Why ?
PLAT154_ALERT_1_G The s.u.'s on the Cell Angles are Equal ..(Note)      0.003 Degree
PLAT180_ALERT_4_G Check Cell Rounding: # of Values Ending with 0 =          3 Note
PLAT186_ALERT_4_G The CIF-Embedded .res File Contains ISOR Records          1 Report
PLAT242_ALERT_2_G Low    'MainMol' Ueq as Compared to Neighbors of       C00X Check
PLAT242_ALERT_2_G Low    'MainMol' Ueq as Compared to Neighbors of       C018 Check
PLAT242_ALERT_2_G Low    'MainMol' Ueq as Compared to Neighbors of       C019 Check
PLAT242_ALERT_2_G Low    'MainMol' Ueq as Compared to Neighbors of       C01B Check
PLAT242_ALERT_2_G Low    'MainMol' Ueq as Compared to Neighbors of       C01D Check
```

```
PLAT242_ALERT_2_G Low    'MainMol' Ueq as Compared to Neighbors of       C01E Check
PLAT434_ALERT_2_G Short Inter HL..HL Contact F008     ..F00M     .       2.82 Ang.
                                                1+x,y,z  =      1_655 Check
PLAT434_ALERT_2_G Short Inter HL..HL Contact F00E     ..F00M     .       2.77 Ang.
                                             -x,1-y,2-z  =      2_567 Check
PLAT720_ALERT_4_G Number of Unusual/Non-Standard Labels ..........         57 Note
              Ag01    Ag02    Ag03    F004    F005    F006    F007    F008
              F009    F00A    O00B    F00D    F00E    F00F    N00G    C00Y
              C00S    H00S    C00Z    N00H    N00I    C01A    C016    H016
              C015    N00L    F00K    F00M    F00N    F00O    F00P    F00Q
              C00T    C013    H013    C014    H014    C017    H017    C011
              H011    C00V    C00U    H00U    C00R    N00C    N00J    C00W
              C00X    C010    F012    C018    C019    C01B    F01C    C01D
              C01E
PLAT860_ALERT_3_G Number of Least-Squares Restraints .............          6 Note
PLAT883_ALERT_1_G Absent Datum for _atom_sites_solution_primary ..     Please Do !
PLAT912_ALERT_4_G Missing # of FCF Reflections Above STh/L=  0.600        294 Note
PLAT933_ALERT_2_G Number of HKL-OMIT Records in Embedded .res File         24 Note
                1 -3 15,   3 -2 13,   4 -1 10,   5 -5 13,   5 -3 11,   5 -2 10,
                5 -1  8,   5 -1  9,   5  0  7,   5  0  8,   6 -5 11,   6 -4 10,
                6 -3  9,   6 -2  7,   6 -2  8,   6 -1  7,   7-11  9,   7 -9  9,
                7 -7 10,   7 -5  9,   7 -4  7,   7 -4  8,   8 -7  8,   8 -5  7,
PLAT941_ALERT_3_G Average HKL Measurement Multiplicity ..........        2.4 Low
PLAT969_ALERT_5_G The 'Henn et al.' R-Factor-gap value ..........      3.569 Note
              Predicted wR2: Based on SigI**2  5.16 or SHELX Weight 16.91
PLAT978_ALERT_2_G Number C-C Bonds with Positive Residual Density.          0 Info
```

---

   0 **ALERT level A** = Most likely a serious problem - resolve or explain
   0 **ALERT level B** = A potentially serious problem, consider carefully
  21 **ALERT level C** = Check. Ensure it is not caused by an omission or oversight
  21 **ALERT level G** = General information/check it is not something unexpected

   3 ALERT type 1 CIF construction/syntax error, inconsistent or missing data
  28 ALERT type 2 Indicator that the structure model may be wrong or deficient
   6 ALERT type 3 Indicator that the structure quality may be low
   4 ALERT type 4 Improvement, methodology, query or suggestion
   1 ALERT type 5 Informative message, check

Validation response form

Please find below a validation response form (VRF) that can be filled in and pasted into your CIF.

```
# start Validation Reply Form
_vrf_PLAT042_lcz062
;
PROBLEM: Calc. and Reported MoietyFormula Strings  Differ     Please Check
RESPONSE: ...
;
_vrf_PLAT213_lcz062
;
PROBLEM: Atom F00O            has ADP max/min Ratio .....        3.7 prolat
RESPONSE: ...
;
_vrf_PLAT220_lcz062
;
```

```
PROBLEM: NonSolvent   Resd 1  F   Ueq(max)/Ueq(min) Range        4.3 Ratio
RESPONSE: ...
;
_vrf_PLAT342_lcz062
;
PROBLEM: Low Bond Precision on  C-C Bonds ...............    0.01455 Ang.
RESPONSE: ...
;
_vrf_PLAT362_lcz062
;
PROBLEM: Short  C(sp3)-C(sp2) Bond  C00Z     - C018     .       1.41 Ang.
RESPONSE: ...
;
_vrf_PLAT906_lcz062
;
PROBLEM: Large K Value in the Analysis of Variance ......      9.146 Check
RESPONSE: ...
;
_vrf_PLAT911_lcz062
;
PROBLEM: Missing FCF Refl Between Thmin & STh/L=    0.600         56 Report
RESPONSE: ...
;
_vrf_PLAT971_lcz062
;
PROBLEM: Check Calcd Resid. Dens.  1.02Ang From Ag02           2.32 eA-3
RESPONSE: ...
;
_vrf_PLAT972_lcz062
;
PROBLEM: Check Calcd Resid. Dens.  0.97Ang From Ag03          -1.61 eA-3
RESPONSE: ...
;
# end Validation Reply Form
```

It is advisable to attempt to resolve as many as possible of the alerts in all categories. Often the minor alerts point to easily fixed oversights, errors and omissions in your CIF or refinement strategy, so attention to these fine details can be worthwhile. It is up to the individual to critically assess their own results and, if necessary, seek expert advice.

PLATON version of 17/09/2025; check.def file version of 17/09/2025

---

duplicate check

No duplication found

Datablock lcz062 - ellipsoid plot

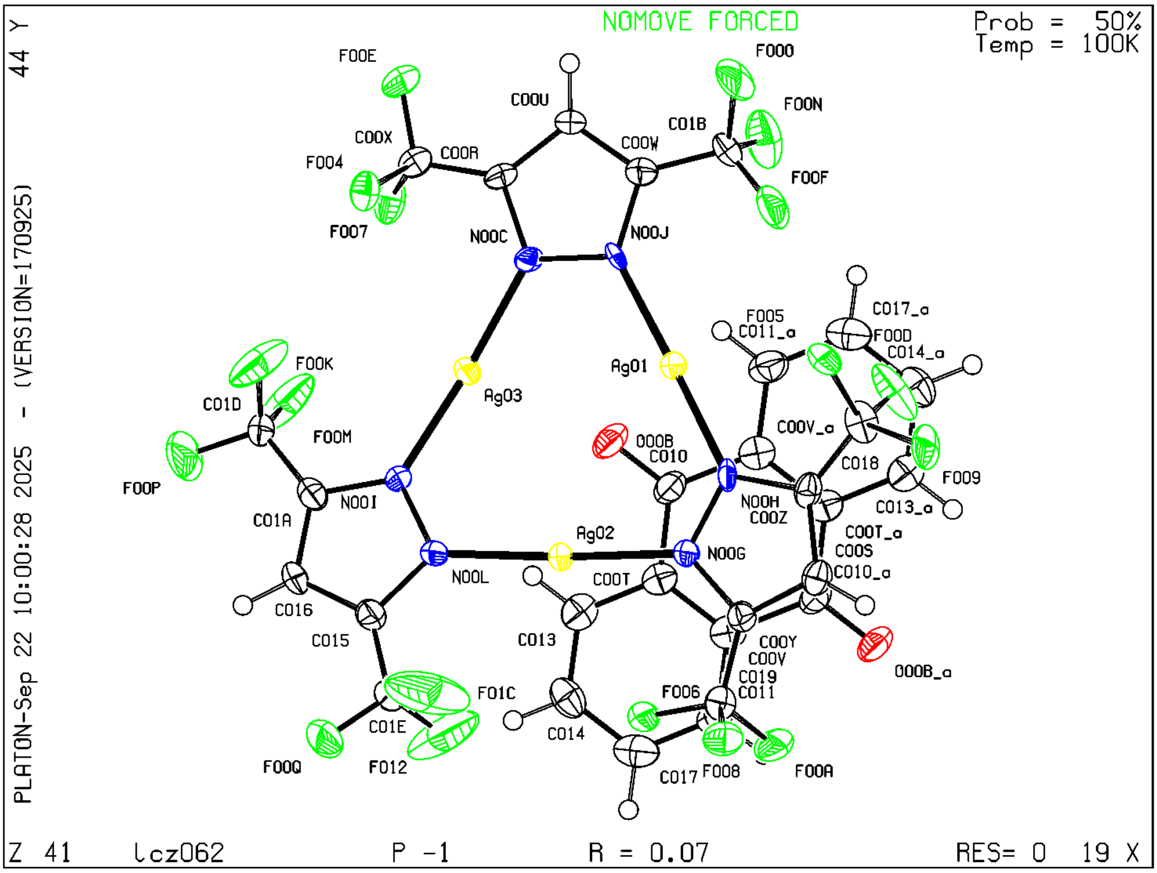

Supplement: Supplementary file 2 — Supporting File 2: anie72462‐sup‐0002‐DataFile.zip. [file ANIE-65-e1218503-s002.zip › supplementary_materials_crystallographic_data submit.zip!/cif_and_checkcif(1-114)/Ag3Pz3_62/Ag3Pz3_62_checkcif.pdf]
